# Electrodegradation of Resorcinol on Pure and Catalyst-Modified Ni Foam Anodes, Studied under Alkaline and Neutral pH Conditions

**DOI:** 10.3390/molecules23061293

**Published:** 2018-05-28

**Authors:** Tomasz Mikolajczyk, Boguslaw Pierozynski, Lech Smoczynski, Wieslaw Wiczkowski

**Affiliations:** 1 Department of Chemistry, Faculty of Environmental Management and Agriculture, University of Warmia and Mazury in Olsztyn, Plac Lodzki 4, 10-727 Olsztyn, Poland; tomasz.mikolajczyk@uwm.edu.pl (T.M.); lechs@uwm.edu.pl (L.S.); 2 Institute of Animal Reproduction and Food Research, Polish Academy of Sciences in Olsztyn, Tuwima 10 Street, 10-748 Olsztyn, Poland; w.wiczkowski@pan.olsztyn.pl

**Keywords:** resorcinol electrodegradation, Ni foam anode, a.c. impedance spectroscopy, HPLC/MS analysis, SEM/EDX analysis

## Abstract

This work reports on the kinetics of electrochemical degradation of the resorcinol molecule, examined on nickel foam-based electrodes in contact with 0.1 M NaOH and 0.5 M Na_2_SO_4_ supporting electrolytes. The electrooxidation of resorcinol was examined on as-received, as well as on Pd-modified, nickel foam catalyst materials, produced via spontaneous deposition of trace amounts of palladium element. Electrochemical (cyclic voltammetry and a.c. impedance) experiments were carried out by means of a three-compartment, pyrex glass electrochemical cell, whereas continuous resorcinol electrooxidation tests were conducted galvanostatically (or potentistatically) with a laboratory-size, single-cell electrolyzer unit. In addition, quantitative determination of resorcinol and its possible electrodegradation products was performed by means of instrumental HPLC: High-Performance Liquid Chromatography/MS: Mass Spectrometry methodology. Also, SEM (Scanning Electron Microscopy) and EDX (Energy Dispersive X-ray spectroscopy) techniques were employed for Ni foam (Pd-modified Ni foam) surface characterizations.

## 1. Introduction

Phenolic compounds are well-known for exhibiting numerous toxic effects on human beings and animals, ranging from mild skin irritation through severe disturbance of the central nervous system [1,2]. Resorcinol is a hydroxy derivative of phenol, one of the most important chemical pollutants present in industrial wastewaters. It is widely used in the production of rubber elements, various specialty chemicals, antioxidants, UV stabilizers, wood additives, dyes, pharmaceuticals, agricultural fertilizers and cosmetics [2,3]. Because of its widespread technological abundance, resorcinol is frequently discharged into the environment as part of industrially generated effluent.

Extensive research activities have shown that electrochemical oxidation is one of the most effective methods to be utilized for the degradation of phenolic compounds. Several catalyst anodes have been investigated for this purpose, including noble/semi-noble and transition metals [3,4,5,6,7]. However, employment of very costly or relatively expensive metals makes this method too expensive for common application, and therefore highly impractical. As a result, in order to make electrochemical degradation of phenolic compounds technologically feasible, one should be looking for baseline electrode materials that are inexpensive, strongly corrosion-resistant in industrial wastewater environments, and highly-modifiable (e.g., by traces of noble/semi-noble metals) [8,9].

**Scheme 1:**
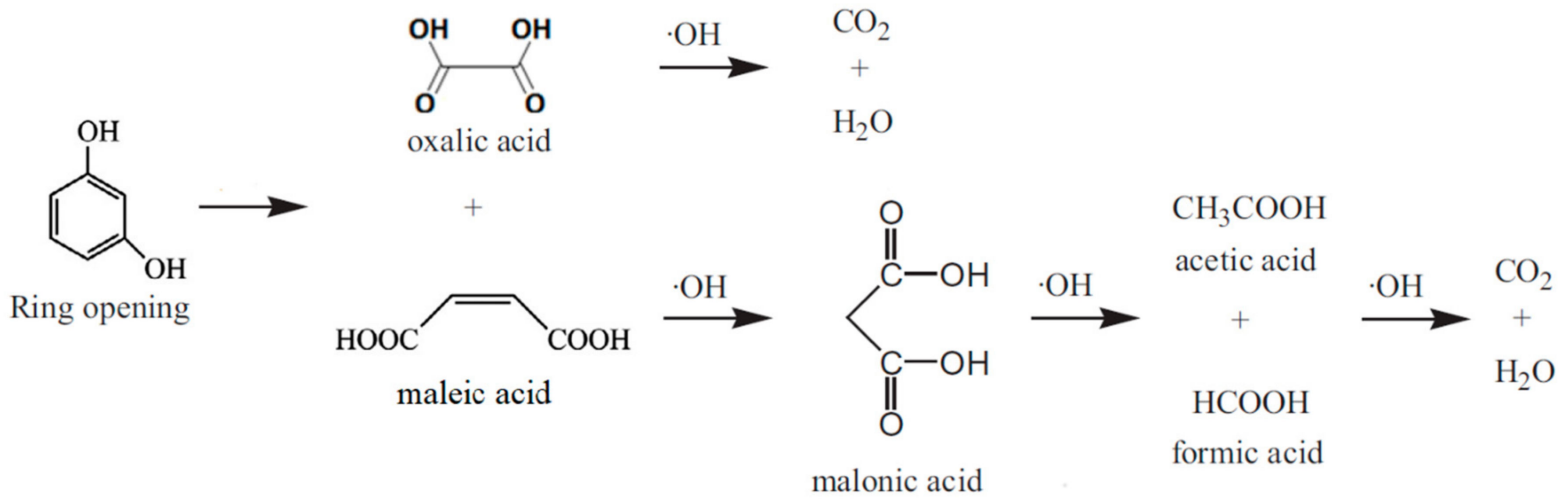
Schematic representation of electrochemical resorcinol degradation processes [6,10].

The process of resorcinol oxidation is usually associated with the conversion of a resorcinol molecule to carboxylic acids and ultimately formation of H_2_O and CO_2_ molecules (a simplified resorcinol electrochemical degradation pathway is shown in Scheme 1 below).

**Scheme 2:**
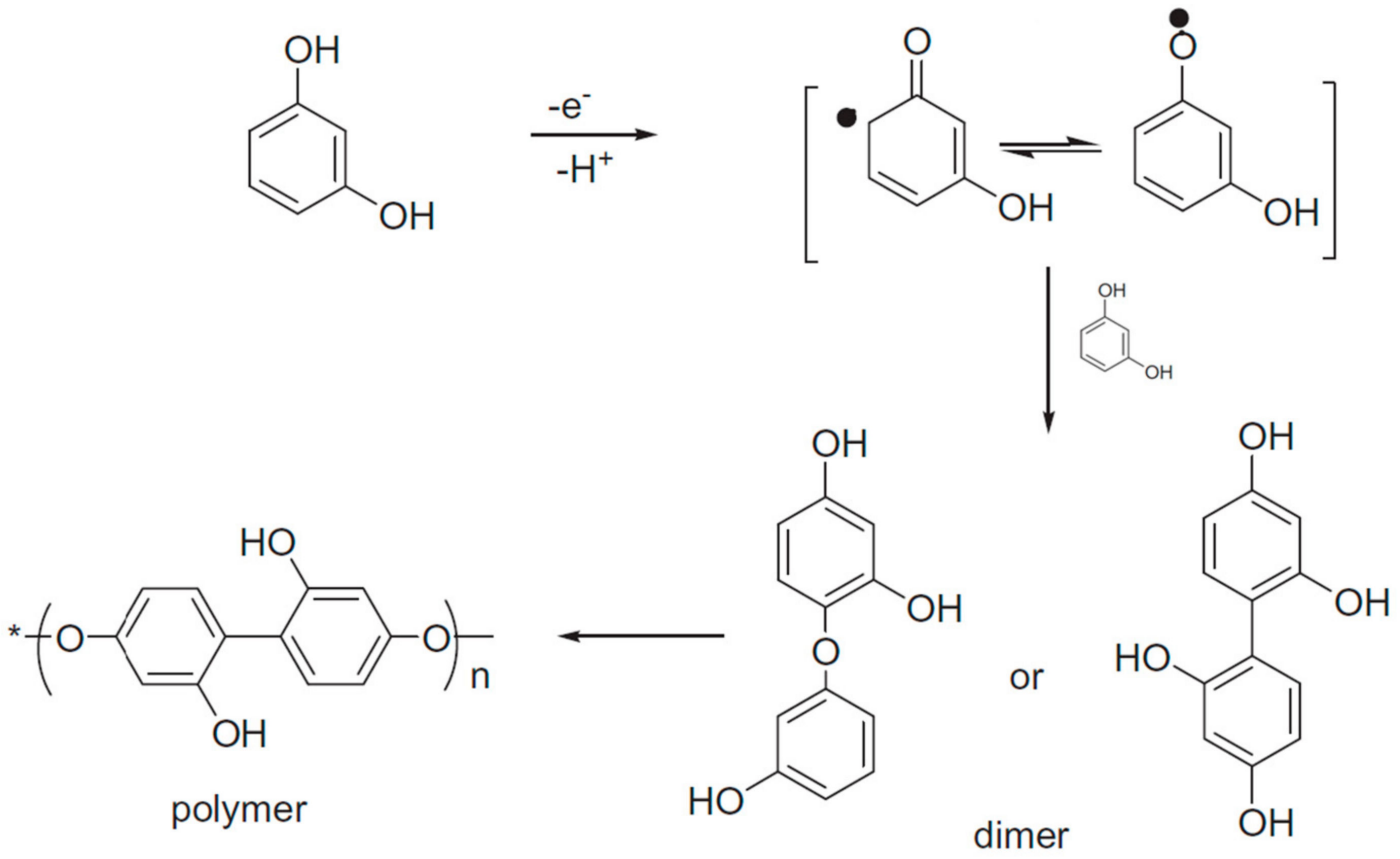
Schematic representation of electrochemical resorcinol polymerization processes [5].

However, in an alternative pathway suggested by Nady et al. (see Reference [5]), the oxidation of resorcinol could partly lead to the formation of polymers. In this model, initially formed radicals react with each other to form dimers (see Scheme 2). The emerged dimers are more easily oxidized than monomers [5], which finally results in oligomer formation, leading to polymer growth causing significant deactivation of the electrode surface.

The purpose of this work was principally concerned with the kinetic aspects of resorcinol electrooxidation and electrosorption reactions, examined on pure and Pd-modified Ni foam electrode materials in 0.1 M NaOH and 0.5 M Na_2_SO_4_ supporting electrolytes. This study is specifically related to some earlier work published from this laboratory, in which Ni-based electrodes were successfully employed to examine the kinetics of phenol electrooxidation reaction [11].

## 2. Materials and Methods 

All solutions used in this work were prepared by utilization of an ultra-pure water purification system from Millipore: Direct-Q3 UV with 18.2 MΩ cm water resistivity. Supporting solutions of 0.1 M NaOH and 0.5 M Na_2_SO_4_ were made up from Merck 99.99% NaOH pellets and Merck 99.99% Na_2_SO_4_ pellets, respectively. Additionally, 0.5 M H_2_SO_4_ solution was prepared from sulphuric acid (SEASTAR Chemicals, Sidney, BC, Canada) to charge a Pd reversible hydrogen electrode (RHE). The resorcinol concentration (Sigma-Aldrich (Saint Louis, MO, USA), >99%) was on the order of 1 × 10^−3^ M.

Two types of electrochemical cells were used during the course of this work. Hence, all kinetic investigations were carried out with a typical, three-compartment Pyrex glass made electrochemical cell, whereas a single-cell electrolyzer unit was employed to perform continuous (8-h long) galvanostatic/potentiostatic resorcinol oxidation tests. Both cells contained three electrodes: a Ni foam-based working electrode (WE), the RHE as reference, and a counter electrode (CE) made from a coiled Pt wire (1.0 mm diameter, 99.9998% purity, Johnson Matthey, Inc., Audubon, PA, USA) and stainless steel, correspondingly.

Nickel foam was delivered by MTI Corporation (purity: >99.99% Ni; thickness: 1.6 mm; surface density: 346 g m^−2^; porosity: ≥95%). Examined electrodes were in two sizes, namely: 1 × 1 cm and 5 × 5 cm (continuous, 8-h long electrolysis trials). Information on the preparation of working electrodes (including Pd catalyst modification) and on all pre-treatments was given in Reference [12]. Nevertheless, it should be stated that for a 1 × 1 cm nickel foam electrode (*ca*. 33.4 mg mass), the electrochemically active surface area was roughly estimated (based on the a.c. impedance-derived double-layer capacitance parameter) in Reference [12] at 13.9 cm^2^. However, as it is very difficult to quantitatively determine the surface area of such a porous entity as a Ni foam electrode, it is, therefore, quite convenient to present the recorded electrochemical results as per the electrode mass. In this work, average values of Ni foam electrode mass were estimated at: 35.8 mg (1 × 1 cm) and 894.2 mg (5 × 5 cm).

All electrochemical measurements were conducted at room temperature by means of the *Solartron* 12,608 W Full Electrochemical System, containing a 1260 frequency response analyzer (FRA) and 1287 electrochemical interface (EI) units. Electrochemical impedance spectroscopy and cyclic voltammetry, as well as continuous, 8-h long galvanostatic/potentiostatic resorcinol electrodegradation experiments, were carried out in this work. For a.c. impedance measurements, the generator provided an output signal of 5 mV and the frequency range was swept between 1.0 × 10^5^ and 0.5 × 10^−1^ Hz. The instruments were controlled by *ZPlot* 2.9 or *Corrware* 2.9 software for Windows (Scribner Associates, Inc., Southern Pines, NC, USA). Usually, three impedance measurements were independently conducted at each potential value on two Ni foam electrodes. Reproducibility of the thus-obtained results was typically below 10%. Data analysis was performed with *ZView* 2.9 (*Corrview* 2.9) software package, where the impedance spectra were fitted by means of a complex, non-linear, least-squares immittance fitting program, *LEVM* 6, written by J.R. Macdonald [13].

Furthermore, selected pre-electrolyzed wastewater samples were subjected to a quantitative assessment of the reaction products/intermediates by means of the combined HPLC/MS analysis. Hence, the analyses were conducted by means of HPLC (LC 20 Prominence, Shimadzu, Japan) system combined with QTRAP 5500 mass spectrometer (AB SCIEX, Concord, ON, Canada), equipped with an ESI ion source, triple quadrupole and an ionic trap. Reaction products were separated by means of XBridge C18 (3.5 µm, 150 × 2.1 mm) chromatographic column (Waters, Milford, MA, USA) at 45 °C for the mobile phase flow of 0.2 mL min^−1^. Both qualitative and quantitative analyses were conducted based on the MRM (Multiple Reaction Monitoring) method. The quantitative analysis was performed through the application of linear calibration curves (R^2^ = 0.993), based on a series of dilutions of external standards. In addition, comparative determination of resorcinol was also carried out based on the bromometric titration method [14,15].

On the other hand, spectroscopic characterization of pure and Pd-modified Ni foam electrodes, before and after resorcinol electrooxidation trials, was performed by means of Quanta FEG 250 Scanning Electron Microscope (SEM), equipped with an Energy-Dispersive X-ray Spectroscopy (EDX) supplement (Bruker XFlash 5010). EDX tests were carried out at an acceleration voltage of 12 kV with a primary intention to confirm the presence of resorcinol polymer on the surface of the examined Ni foam electrodes.

## 3. Results and Discussion

### 3.1. EDX and SEM Characterizations of Nickel Foam and Pd-Modified Nickel Foam Electrodes

**Figure 1:**
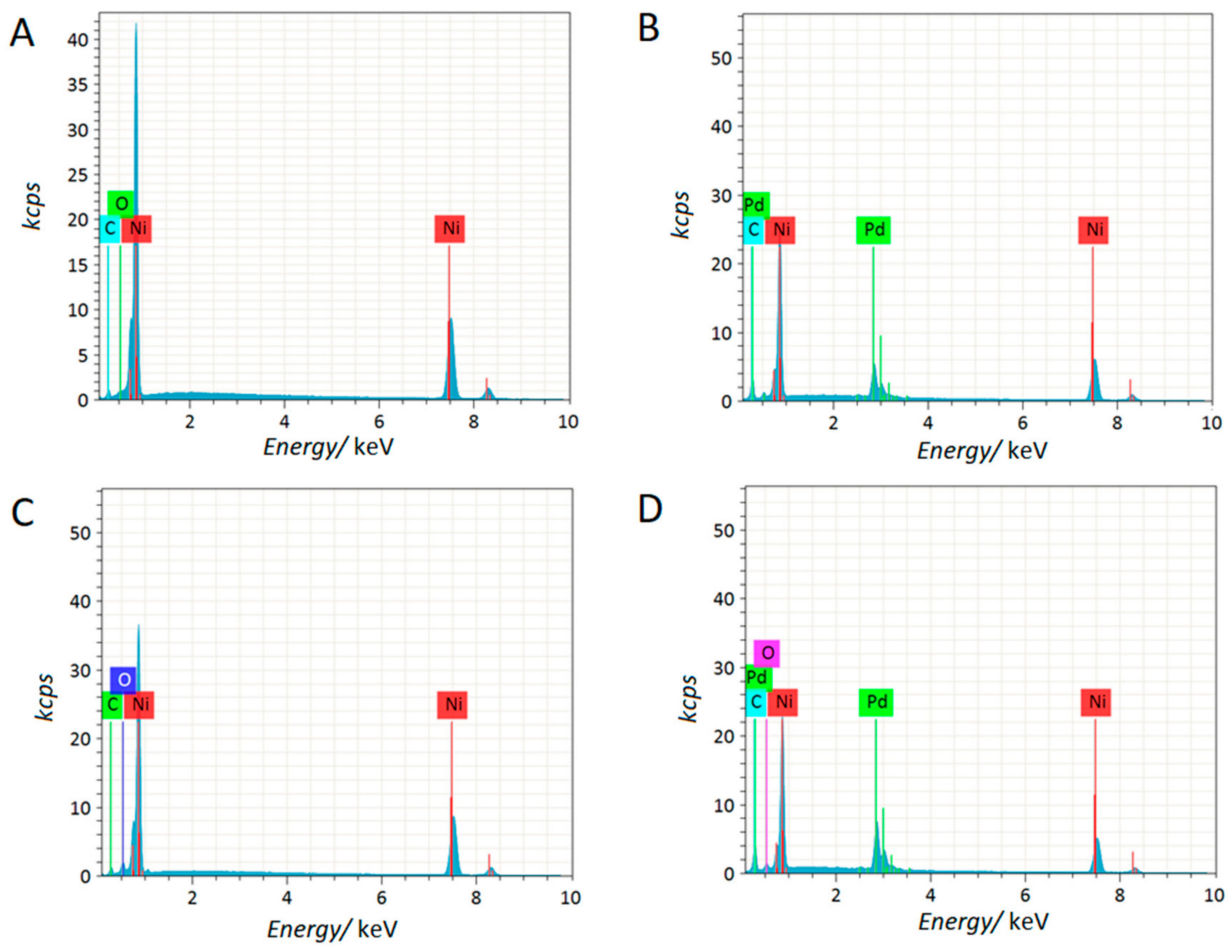
(**A**) EDX spectrum carried out at an acceleration voltage of 12 kV and the working distance of 9.8 mm for fresh Ni foam electrode; (**B**) as in (**A**), but for Pd-modified Ni foam; (**C**,**D**) as in (**A**,**B**) respectively, but after electrochemical examinations.

Figure 1A–D and Table 1 below present EDX spectra and EDX surface compositions for fresh and the Pd-modified Ni foam electrodes, recorded prior to and after resorcinol electrooxidation experiments have been carried out. Here, the most important observation is that surface electrodegradation of resorcinol led to a radical increase of the recorded average wt. % of carbon element (by ca. 1.7× and 1.5× for pure and the Pd-modified nickel foam electrodes, respectively), as compared to unexamined nickel foam electrodes.

In addition, a similar trend was also observed for the surface average content of oxygen, which after electrochemical examination rose by about 8.7× and 4.0× for the unmodified and the Pd-activated nickel foam electrodes, respectively (see Table 1 for details). Considerable increase of the C content for the resorcinol-electrooxidized Ni foam electrodes is in line with the pathway proposed by Nady et al. [5], which involved the surface formation of strongly-bonded polymeric species. However, the latter could also be supported through the observation of surface-adsorbed, yellowish deposit on the nickel foam electrode upon continuous resorcinol electrooxidation reaction. Furthermore, radically rising surface oxygen content is a primary result of simultaneous surface Ni foam oxidation process.

**Figure 2:**
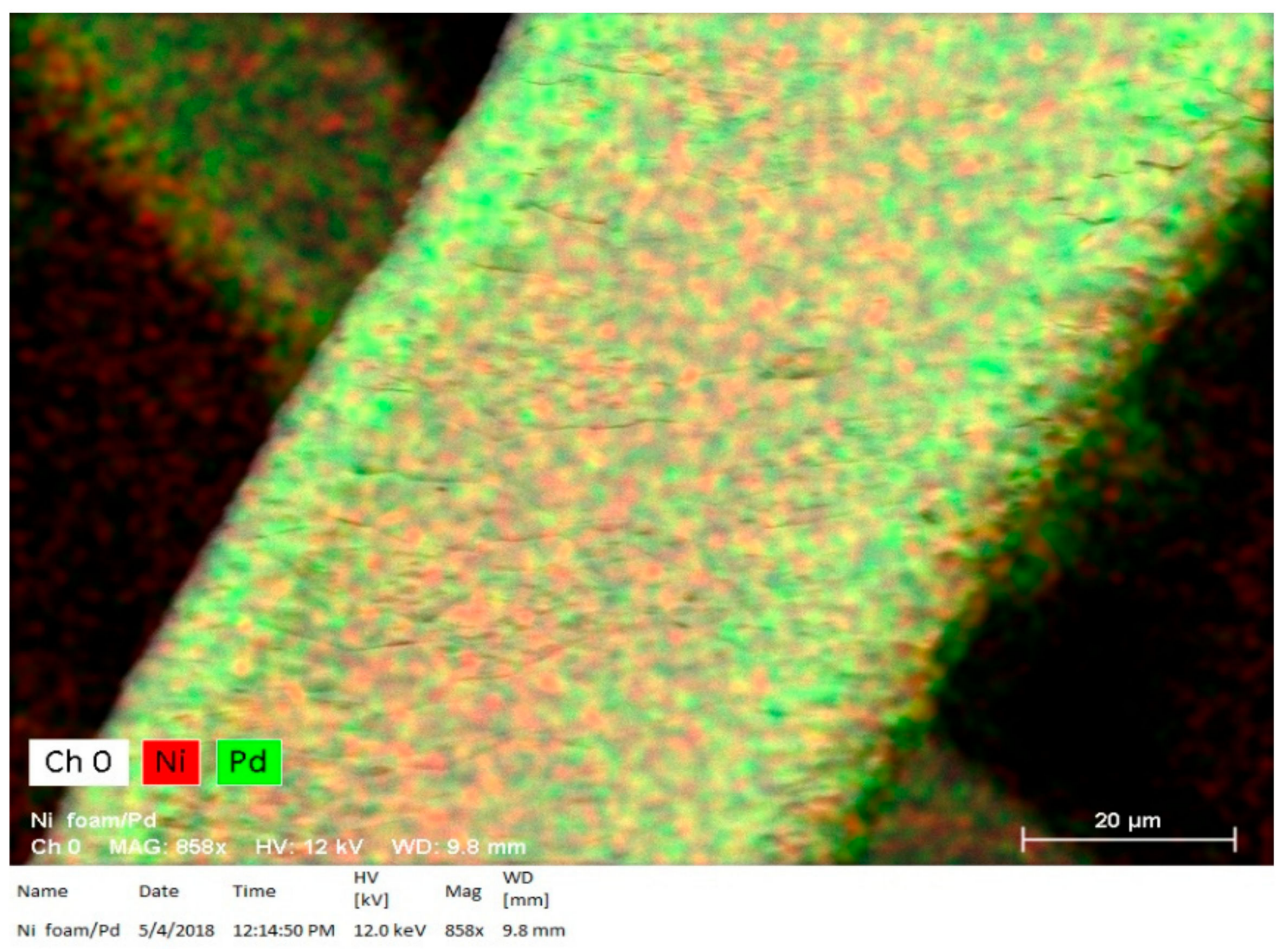
EDX elemental mapping for SEM micrograph picture of a fresh Pd-modified Ni foam electrode taken at 858× magnification, an acceleration voltage of 12 kV and the working distance of 9.8 mm.

Finally, an average content of Pd element (uniformly distributed over the nickel foam surface, see Figure 2) for the palladium-activated nickel foam electrodes was derived by a weighing method at ca. 0.1–0.2 wt. %. However, as for the EDX procedure, the depth of surface penetration (and ultimately the resultant elemental composition) is strongly dependent on the employed acceleration voltage, the EDX-based recorded surface Pd composition (Table 1) is significantly higher than that derived based on the bulk (weighing method) analysis.

### 3.2. Electrochemical Characteristics of Resorcinol Oxidation Reaction

**Figure 3:**
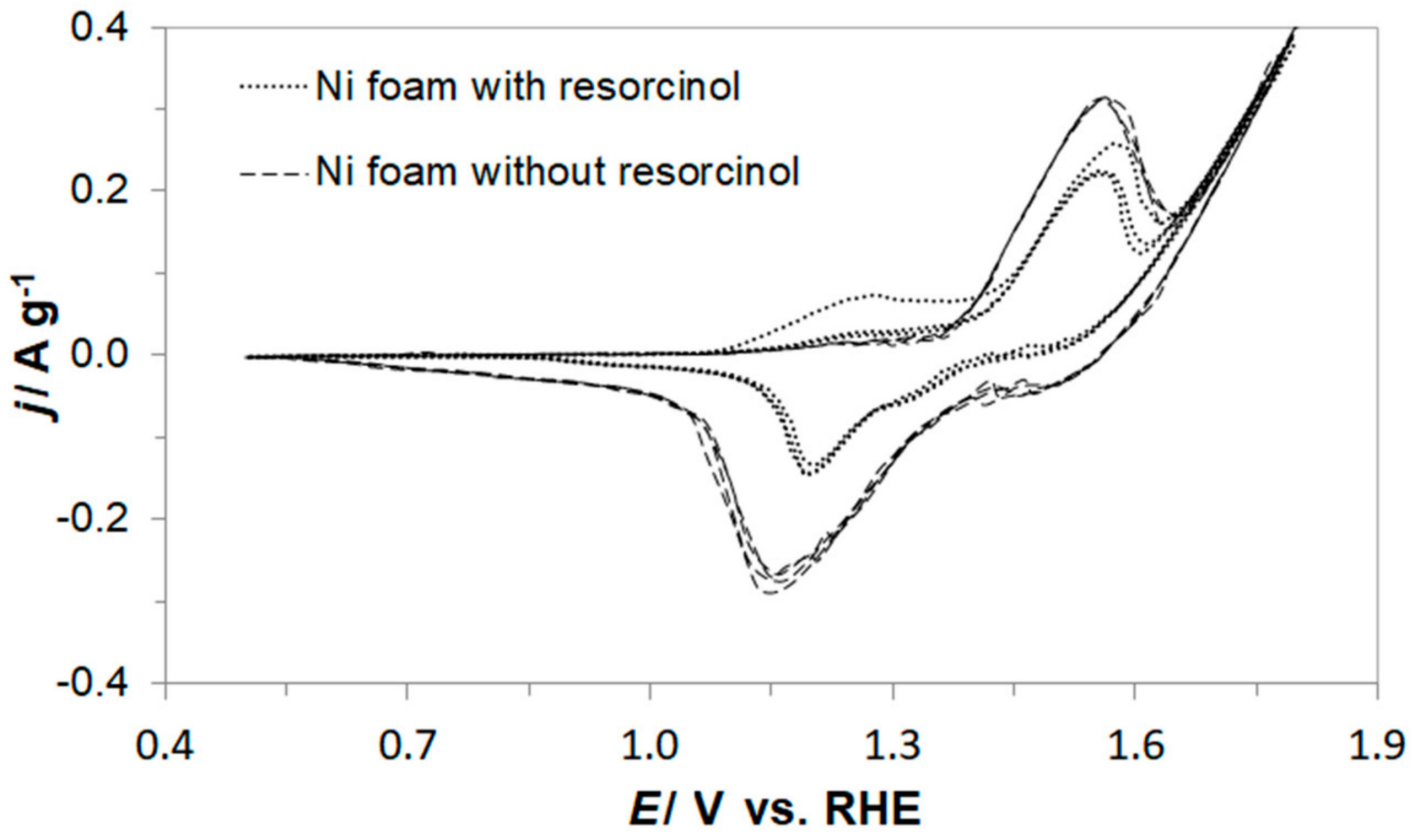
Cyclic voltammograms recorded on Ni foam electrode surface in 0.1 M NaOH supporting solution, carried out in the absence and presence of resorcinol (at 1 × 10^−3^ M), at a sweep-rate of 50 mV s^−1^.

The cyclic voltammetric behavior of Ni foam electrode in contact with 0.1 M NaOH supporting solution, in the absence and presence of resorcinol (at 1 × 10^−3^ M), is shown in Figure 3, below.

In the absence of resorcinol, an anodic oxidation peak could be observed in the CV profile over the potential range: 1.35–1.60 V vs. RHE, which illustrates the process responsible for the formation of NiOOH species from Ni(OH)_2_ [16,17]. On the other hand, in the presence of resorcinol, an initial oxidation peak appears over the potential range ca. 1.10–1.40 V. This anodic peak is associated with the oxidation process of resorcinol [3,5]. Then, as for the resorcinol-free NaOH solution, a second anodic peak is observed for the potential span: 1.40–1.60 V vs. RHE. Subsequently, upon a reverse voltammetric scan, a cathodic peak emerges over the potential range ca. 1.00–1.40 V and 1.10–1.40 V in the absence and presence of resorcinol, respectively. This peak is associated with the reduction of Ni(II) oxidation products [18]. Most importantly, in comparison to the resorcinol-free profile, the CV profile recorded in the presence of resorcinol is characterized by a significant diminution of the voltammetric charge (Figure 3), which strongly supports the concept of partial surface coverage by the resorcinol electrodegradation polymer product.

**Figure 4:**
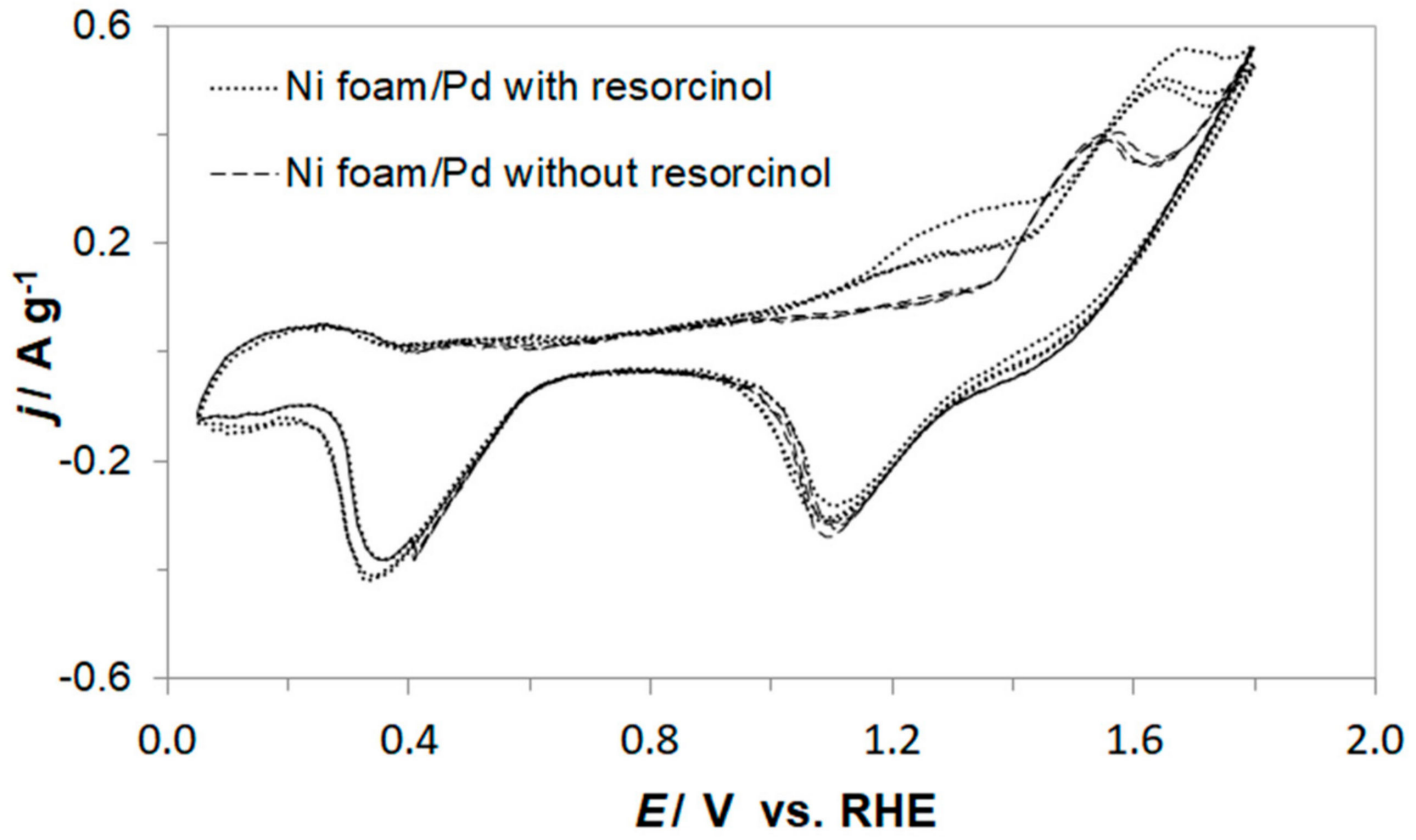
Cyclic voltammograms recorded on Pd-modified Ni foam electrode surface in 0.1 M NaOH supporting solution, carried out in the absence and presence of resorcinol (at 1 × 10^−3^ M), at a sweep-rate of 50 mV s^−1^_._

Similar voltammetric behavior, examined under analogous experimental conditions, was recorded on the Pd-modified Ni foam electrode (see Figure 4), where apparent anodic resorcinol oxidation peak is observed over the potential range: 1.00–1.50 V. Also, respective peaks corresponding to the processes of formation and reduction of nickel oxyhydroxide could clearly be identified in the voltammetric profiles of this Figure. An additional cathodic peak, which refers to the reduction of Pd surface oxides (formed at more positive potentials) is observed over the potential range: 0.25–0.65 V vs. RHE [19]. Finally, the voltammetric feature recorded over the potential range: 0.05–0.30 V could be assigned to the reversible H UPD (Hydrogen Underpotential Deposition) phenomenon taking place at palladium (see Refs. [20,21] for details on this process).

**Figure 5:**
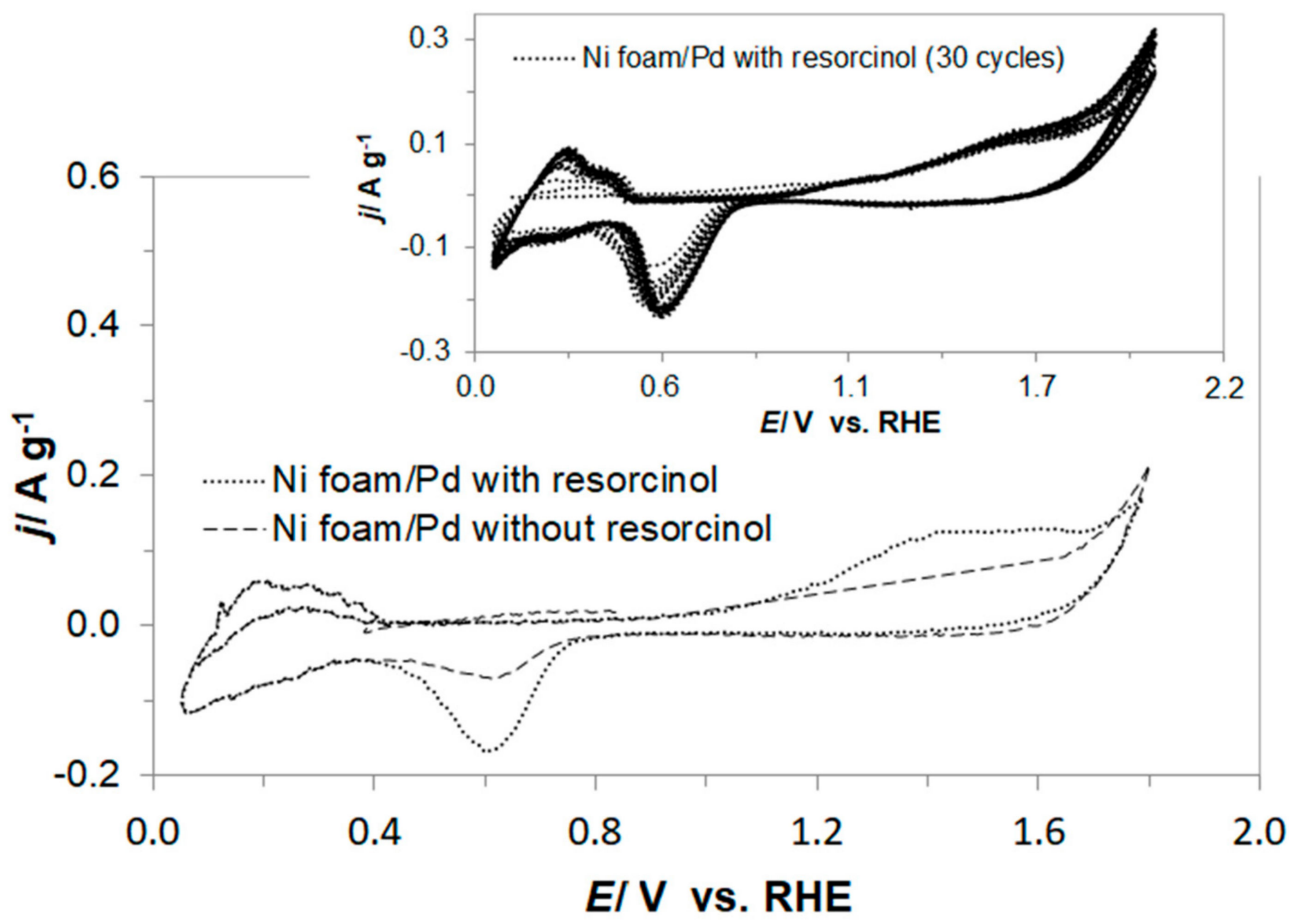
Cyclic voltammograms recorded on Pd-modified Ni foam electrode surface (inset presents 30 continuous CV sweeps) in 0.5 M Na_2_SO_4_ supporting solution, carried out in the absence and presence of resorcinol (at 1 × 10^−3^ M), at a sweep-rate of 50 mV s^−1^_._

On the surface of unmodified nickel foam, no resorcinol electrodegradation was noticed (a respective CV profile is not shown here). It could then be assumed that significant presence of oxyhydroxide layer (considerably impeded under neutral pH conditions) on the Ni foam surface is necessary in order to commence the process of resorcinol electrooxidation in the absence of Pd metal catalyst. Therefore, the CV profile for the Pd-modified Ni foam electrode (Figure 5) is characterized by a single, broad anodic peak over the high potential range. This peak (1.20–1.70 V vs. RHE) corresponds to the resorcinol electrooxidation reaction. Other features observed in the potential span 0.00–0.80 V are analogous to those referred to in Figure 4 above.

**Figure 6:**
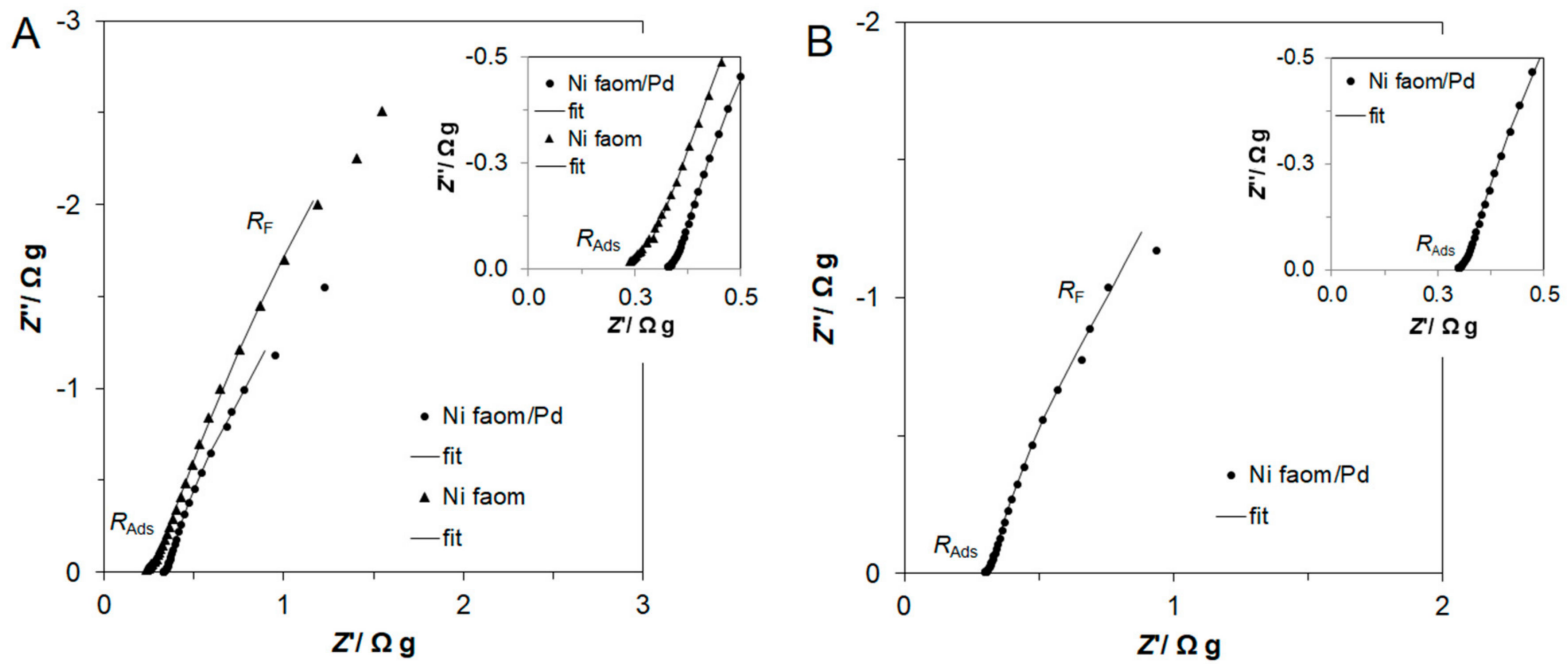
(**A**) Complex-plane impedance plots for resorcinol electrooxidation on Ni foam and Pd-modified Ni foam electrode surfaces in contact with 0.1 M NaOH solution, recorded at room temperature for a potential value of 1200 mV vs. RHE. The inset presents a magnification of the semicircle assigned to *R*_Ads_ parameter for both electrodes. The solid lines correspond to a representation of the data according to equivalent circuit model shown in Figure 7; (**B**) as in (**A**), but for Pd-modified Ni foam electrode in contact with 0.5 M Na_2_SO_4_ solution.

Furthermore, the a.c. impedance kinetic characterization of the resorcinol electrooxidation process on the unmodified Ni foam surface (electrode I) and the Pd-modified Ni foam (electrode II) in NaOH solution, and the palladium-modified Ni foam, but examined in Na_2_SO_4_ supporting electrolyte (electrode III), is shown in Figure 6A,B, and Table 2.

**Figure 7:**
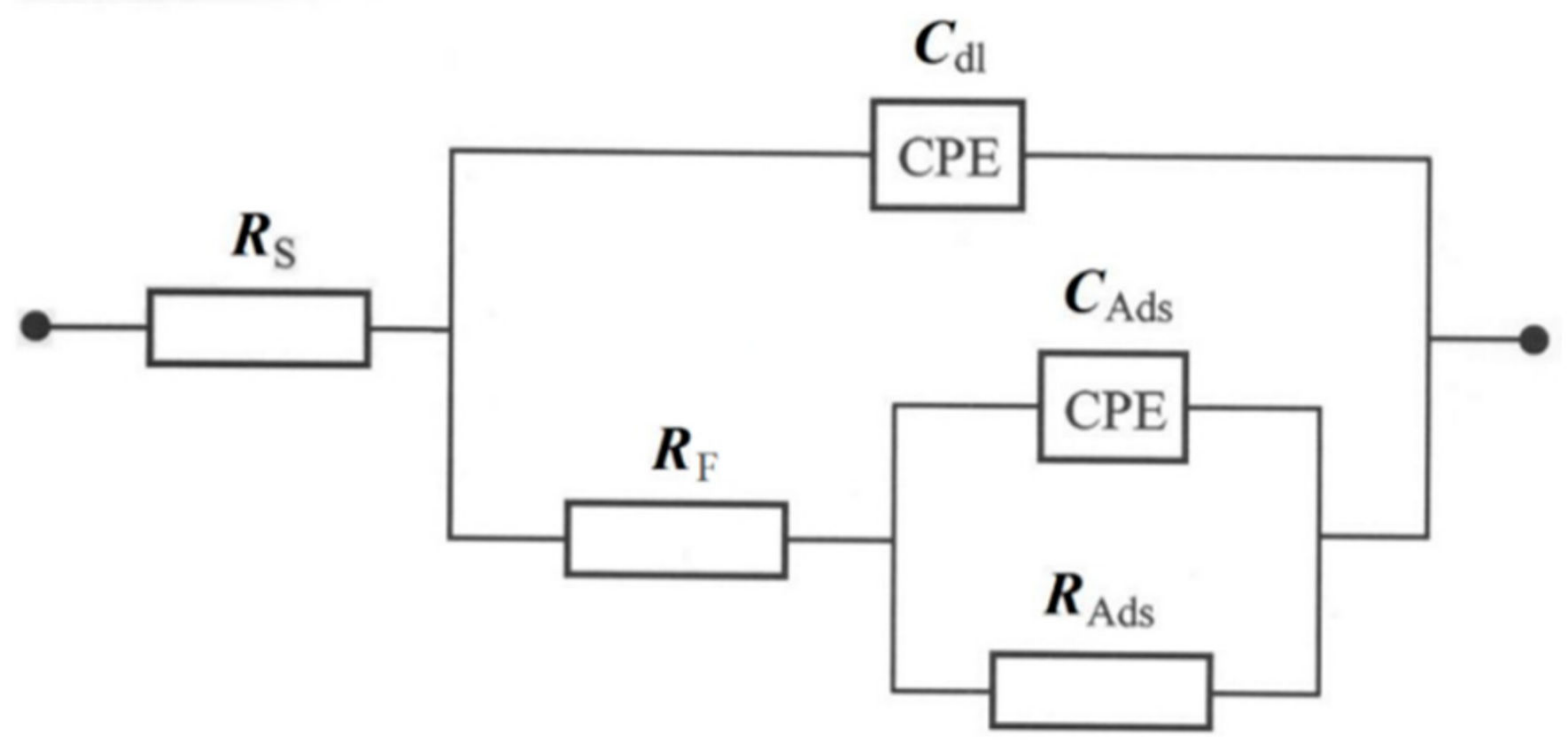
Equivalent circuit model for electrooxidation of resorcinol on Ni foam-based electrodes. The circuit exhibits a Faradaic charge-transfer resistance, *R*_F_, the rate of adsorption (or desorption) of resorcinol (*R*_Ads_) and the component that contains the contribution from the surface coverage of the adsorbed intermediate, *C*_Ads_ (represented here as the constant phase element—CPE). The above components are in a parallel combination with the double-layer capacitance, *C*_dl_ (also shown as the CPE), jointly in series with an uncompensated solution resistance, *R*_S_.

Thus, for all examined electrodes (I, II, III), the Nyquist impedance spectra recorded over the studied potential range (1100–1400 mV/RHE) exhibited two somewhat depressed partial semicircles, clearly distinguishable in the impedance plots. Here, a high-frequency semicircle refers to the process of resorcinol electrosorption (see insets to Figure 6A,B), while a low/intermediate-frequency arc corresponds to the Faradaic resorcinol oxidation reaction (see Figure 7 for equivalent circuit model) [11,22]. Hence, the minimum of the adsorption resistance, *R*_Ads_, parameter for all electrodes oscillates around 0.06–0.12 Ω g. On the other hand, the recorded charge-transfer resistance, *R*_F_ parameter for all three examined electrodes tends to rise upon augmentation of the probing potential (e.g., 10.38, 13.63 and 21.68 Ω g were recorded in Table 2 over the potential range: 1100–1300 mV for the electrode denoted as I). The above could be explained in terms of significant obstruction of the Faradaic electrodegradation process, as the surface blocking by the polymeric layer intensifies. In addition, the latter observation could strongly be supported by continuous and radical reduction of the *C*_dl_ and *C*_Ads_ capacitance parameters with rising working electrode potential (see Table 2 for details).

**Figure 8:**
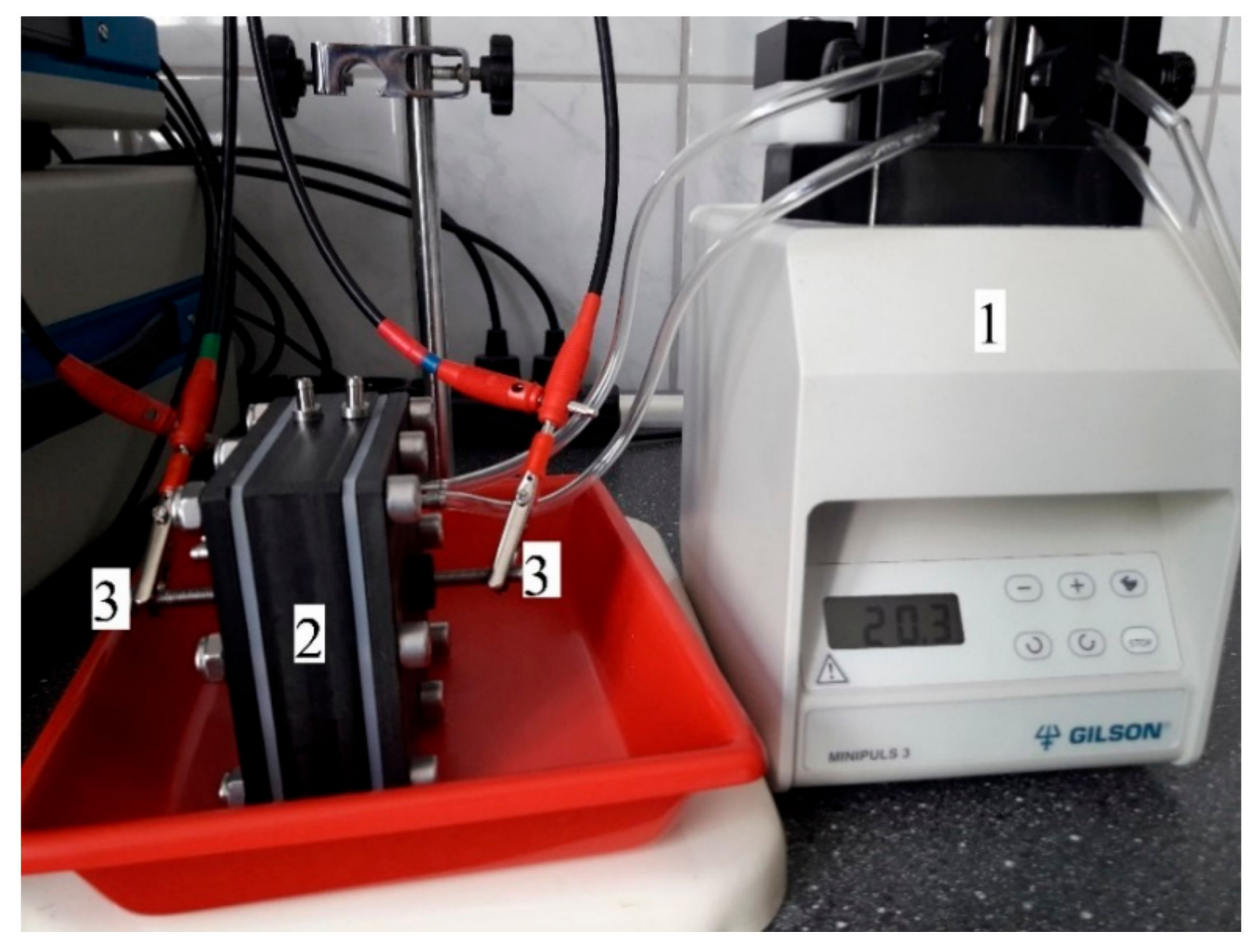
A single-cell electrolyzer unit employed for the 8-h electrolysis trials: 1—Gilson Minipuls 3 peristaltic pump; 2—electrolyzer unit; 3—electrical connections.

Additionally, the 8-h-long galvanostatic (0.1 M NaOH, electrodes I and II) and potentiostatic (0.5 M Na_2_SO_4_, electrode III) electrolysis trials (Figure 8) were carried out in order to determine the rate of resorcinol removal (for an initial concentration of 1 × 10^−3^ M) from synthetically made wastewater solutions. The anodic current for the galvanostatic mode was set at 50 mA, which referred to a current density of ca. 0.14 mA cm^−2^. On the other hand, the potential for the potentiostatic measurement was fixed at 1.5 V vs. RHE (below the potential for nickel dissolution in neutral pH) with current-density ranging from 0.01 to 0.03 mA cm^−2^.

**Figure 9:**
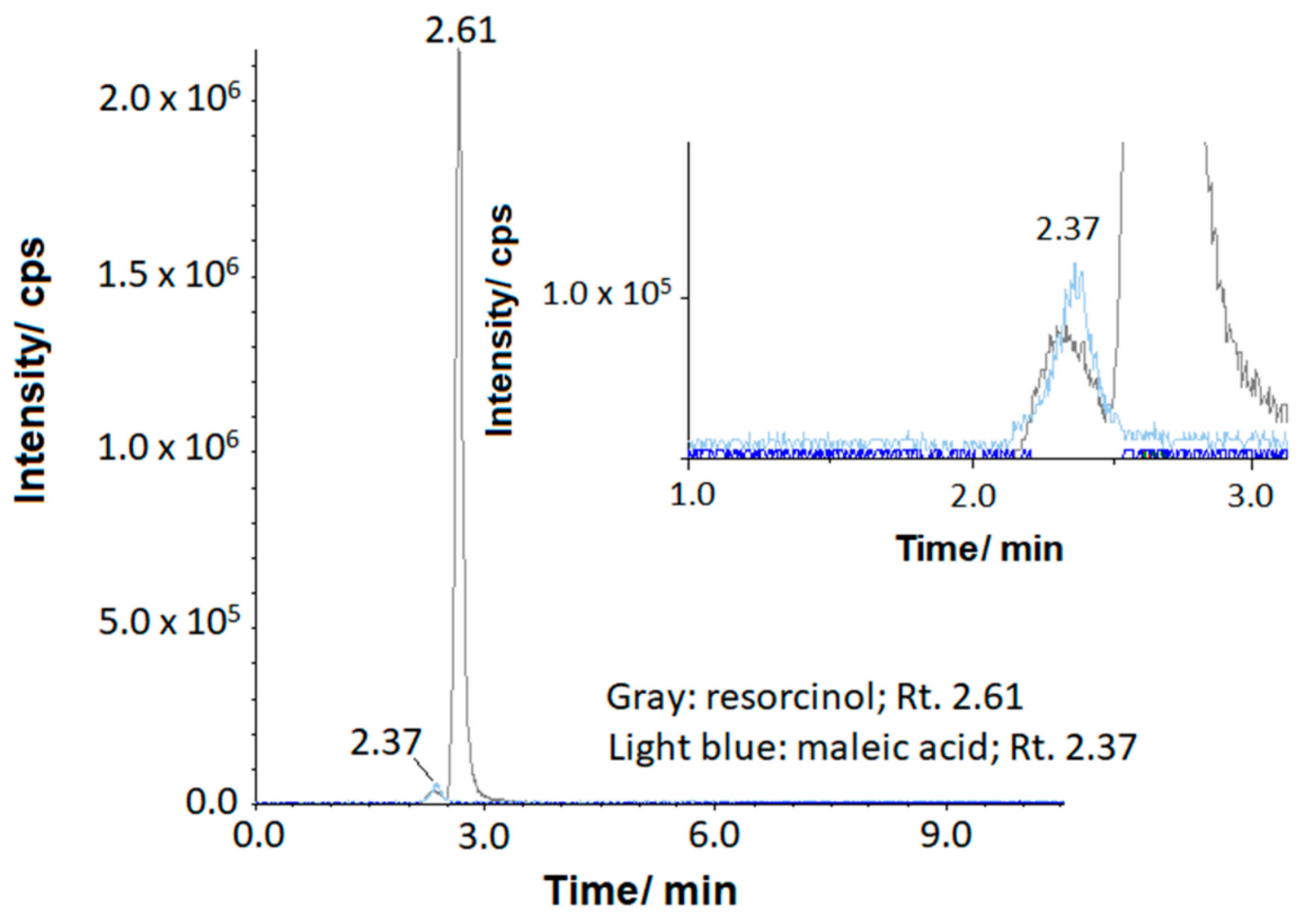
HPLC chromatograms recorded for resorcinol-based, electrolyzed synthetic (Na_2_SO_4_) wastewater solution, exhibiting detected maleic acid intermediate (inset).

Thus, based on the performed bromometric titratium analysis, it could qualitatively be stated that final concentrations of resorcinol in the electrolyzed wastewater samples after the 8-h electrodegradation trials became reduced by at least 50% from an initial resorcinol content for all the examined electrolysis runs. Moreover, the results of HPLC/MS analysis (produced based on short, 2-h electrolysis experiments) suggest that the resorcinol electrodegradation process leads to a breakdown of the aromatic ring and the formation of carboxylic acids, and CO_2_ as its key products. The above is evidenced by the absence of hydroquinone or benzoquinone species in the recorded chromatograms. Actually, maleic acid was the only identified byproduct of the resorcinol electrooxidation process (see as an example Figure 9 below). Its recorded concentration came to 1.6 × 10^−6^ M with a final resorcinol content on the order of 8.7 × 10^−4^ M.

## 4. Conclusions

Nickel foam-based materials are suitable anode catalysts for successfully performing resorcinol electrochemical degradation under alkaline and neutral pH conditions. The process of resorcinol oxidation on the surface of nickel foam leads simultaneously to a destruction of the aromatic ring (to generate carboxylic acids, e.g., maleic acid and CO_2_ products) along with the formation of strongly surface-bonded polymeric layer, which then severely inhibits the processes of resorcinol electrosorption and its Faradaic electrooxidation on the catalyst surface.

The above was proven through the application of cyclic voltammetry, as well as a.c. impedance spectroscopy experiments. Both surface adsorption and oxidation steps were clearly discernible within the recorded Nyquist impedance spectra, where the former process was found to proceed significantly faster (over 150× for the potential of 1300 mV) than the latter one. Finally, employed combined HPLC/MS methodology allowed for a quantitative determination of resorcinol and maleic acid (1.6 × 10^−6^ M), as the reaction intermediate. Most importantly, it excluded the presence of aromatic byproducts, such as: benzoquinone or hydroquinone in the electrolyzed samples of wastewater.

## Autor Contributions

T.M. designed and ran the experiments and prepared the draft version of the manuscript; B.P. oversaw operation of the experiments and co-prepared the final version of the manuscript; L.S. discussed the experimental results; W.W. ran and analyzed the HPLC/MS experiments.

## Figures and Tables

**Table 1:** EDX-derived surface elemental compositions for Ni foam and Pd-modified Ni foam samples.

Elements	Measurements	A *	B *	C *	D *
Ni/%	1	94.05	77.50	87.86	63.80
	2	93.87	70.86	85.56	73.36
	3	95.01	72.36	88.73	74.27
C/%	1	5.39	3.88	9.30	5.70
	2	5.59	3.36	9.31	5.61
	3	4.83	3.97	8.24	5.22
O/%	1	0.56	0.78	2.83	2.40
	2	0.54	0.45	5.13	2.51
	3	0.16	0.56	3.03	2.40
Pd/%	1	—	17.00	—	28.10
	2	—	25.33	—	18.52
	3	—	23.11	—	18.11

* symbols as in Figure 1.

**Table 2:** Resistance and capacitance parameters for the processes of resorcinol electrosorption and electrooxidation (at 1 × 10^−3^ M) on Ni foam (**I**) and Pd-modified Ni foam (**II**) electrode surfaces in contact with 0.1 M NaOH solution, and Pd-modified Ni foam electrode (**III**) surface in contact with 0.5 M Na_2_SO_4_ solution, obtained by fitting the equivalent circuit shown in Figure 7 to the experimentally obtained impedance data (dimensionless φ_1_ and φ_2_ parameters, which determine the constant phase angle in the complex-plane plot (0 ≤ φ ≤ 1) of the CPE circuit, varied between 0.35 and 0.87).

*E*/mV	*R*_Ads_/Ω g	*C*_Ads_/µF g^−1^ s^φ1−1^	*R*_F_/Ω g	*C*_dl_/µF g^−1^ s^φ2−1^
Electrode I				
1100	0.19 ± 0.01	64,157 ± 894	10.38 ± 0.67	91,865 ± 8543
1200	0.11 ± 0.01	54,892 ± 1762	13.63 ± 0.68	65,533 ± 8061
1300	0.12 ± 0.01	54,762 ± 679	21.68 ± 1.94	52,718 ± 7117
**Electrode II**				
1100	0.20 ± 0.01	300,890 ± 1866	3.76 ± 0.05	245,411 ± 1006
1200	0.06 ± 0.00	142,843 ± 1243	11.43 ± 0.34	201,937 ± 666
1300	0.07 ± 0.00	138,788 ± 1485	11.63 ± 0.89	92,558 ± 324
1400	0.44 ± 0.09	87,120 ± 13,168	29.11 ± 2.10	31,699 ± 3828
**Electrode III**				
1100	0.21 ± 0.01	35,251 ± 325	9.32 ± 0.05	32,625 ± 108
1200	0.07 ± 0.00	32,563 ± 243	11.62 ± 0.31	29,328 ± 356
1300	0.08 ± 0.00	30,890 ± 177	11.45 ± 0.89	24,124 ± 89
1400	0.32 ± 0.07	28,545 ± 9512	20.67 ± 1.72	10,359 ± 2576
